# Narrative of a Voyage Round the World, &c.

**Published:** 1836-01-01

**Authors:** 


Narrative of a Voyage round the World, &c. &c. By *T. B. Wilson*, M.D. R.N. Octavo. Sherwood, 1835.

A book like this, written in the most classic style, and abounding in adventures and perilous escapes, would have excited universal attention two centuries ago, or even much later. But now, a "voyage round the world" is no more than a voyage across the Atlantic in the days of our forefathers. The mere circumnavigation of the globe, however, forms but a small part of the interest excited by Dr. Wilson's narrative. It is little, if at all, inferior in this respect, to the celebrated narrative of Captain Bligh, to which, indeed, it bears a great similitude, except that it is much better written. Our reasons for going out of our usual course to notice this volume, will shortly appear, and, we hope, prove valid. It will afford a striking illustration of an important principle of hygiene, which is not sufficiently attended to in medicine.

Dr. Wilson has been eight voyages to New South Wales, in charge of convicts, and has been twice or thrice shipwrecked in that time. In the year 1829, after landing his convicts at Sydney, he re-embarked for Batavia, in the ship called the "Governor Ready;" and, on the 17th of May, the ship struck with such force on a coral reef, near Murray's Island, that the rock penetrated her bottom, and, in a few minutes the water was up to the lower deck! They had now 900 miles, at least, to traverse in open boats, and through a most perilous navigation. It was almost exactly in the same course as that pursued by the launch of "Bounty Bligh." But Dr. Wilson's crew were exposed to more perils than the officers of the Bounty, who had a strong boat to bear them over the pathless deep. We cannot dwell on the various "hair-breath escapes" of the boats, in this long voyage through innumerable rocks and shoals. One scene will be sufficient. One evening a hurricane came on, and the crew of the jolly-boat, being frightened, implored to be taken into the long-boat, already labouring with her cargo, and very leaky. At first they resisted this appeal, but at length complied, at the imminent risk of all their lives. The crew of the jolly-boat no sooner came close to the long-boat, than they made a rush on board the latter, and nearly upset her! They were now in great danger, and could hardly keep the boat before the wind; "when, about nine o'clock, a heavy sea, whose death-denoting sound still lingers in my ears, rolled over the larboard quarter, and filled the boat!" For a moment they were paralyzed, believing that they were going to the bottom; but, finding the boat still floated, they managed to bale out the water. They had scarcely cleared her, when again they were swamped—and again preserved from a watery grave! After a series of miraculous escapes they reached Timor, the Island where Captain Bligh found refuge after his adventurous course! Here they met with some acquaintances, and one of these was so struck with the wan and altered visage of Dr. Wilson, that he burst into tears while surveying the living remains of his friend!! Now, during the whole of this long series of disasters, starvation, and exposure, night and day, to storms and rains in open boats, they lost not a man—nor had they a single instance of sickness! Some of them, indeed, died afterwards, when the toils and dangers were over, and when they had food, raiment, and shelter from the elements. The principle which this illustrates is one which we have deeply studied, and which will shortly be laid before our readers. It is this:—that activity of body and excitement—even painful excitement—of mind, will counteract, in the most surprizing manner, the various causes, moral and physical, of diseases. We have collected, for another occasion, a great many illustrations of this principle, drawn from ancient and modern history. The shipwreck so beautifully narrated by Dr. Wilson shall have a place on the list. We shall here only briefly allude to one or two others.—One of the earliest on record, is the celebrated retreat of Xenophon with the ten thousand Greeks, after the fall of Cyrus on the plains of Cunaxa. This band was left, without commanders, money, or provisions, to traverse a space of *twelve hundred leagues*, under constant alarms from the attacks of barbarous and successive swarms of enemies. They had to cross rapid rivers, penetrate gloomy forests, drag their weary way over vast and burning deserts, scale the summits of rugged mountains, and wade through deep snows and pestilent morasses, pursued by implacable foes! It does not appear that they lost a single man *by sickness* during the whole of this march. Xenophon is often very minute in his statements of the losses they sustained. He even describes the individual cases, and the names of the individuals wounded. Only two instances of sickness are put on record—one a sort of *bulimia*, or canine appetite, produced by the cold of the snow among the Armenian mountains; but it did not prove fatal. The other was an illness of 24 hours' duration, which was general throughout the army, the consequence of indulgence in a kind of honeycomb, which they found in great abundance in one part of Armenia. It produced vomiting and purging amongst those who ate freely; but a kind of drunken delirium in those who ate sparingly.*

As the Greeks lost only 500 men during the whole of this march, and none are mentioned as dying, except by wounds, we may fairly conclude that the

---

* Sir Charles Bagot informed the Editor that he experienced the same thing, after eating honey in some of the mountainous parts of America.

*incessant activity of body, and vigilance of mind,* preserved the ten thousand Greeks from all kinds of disease during this unparalleled retreat.

It is not a little remarkable that Xenophon himself discovered, or at least anticipated, this very PRINCIPLE, at the moment when it was most needed. In the stupor which came over the Greek army, when they saw their Generals butchered by the treacherous Tissaphernes, and themselves surrounded by ruthless enemies, Xenophon, in his address to some of his brother-leaders, says:—"The soldiers have, at present, nothing before their eyes but misfortunes—if any one can TURN THEIR THOUGHTS INTO ACTION, it would greatly encourage them." Here, then, is the very PRINCIPLE itself, happily conceived, and most promptly acted on, by the Athenian General! He tried, and with success, to convert the torpor of despair into the energy of desperation—urging his men to prefer DEATH, in the sanguinary but brief conflict with the enemy, to the *protracted tortures* that would inevitably be the consequence of capture. Then it was that the tents were burnt—the carriages destroyed—the sumpter-horses slaughtered—and every unnecessary incumbrance, except the sword, abandoned. During 215 days of almost uninterrupted march—generally in the face of the enemy—often between two enemies, and engaged in front and rear at the same time, the army lost not one man by SICKNESS!! Here, then, the physical causes of illness were numerous, and the depressing passion of FEAR was necessarily blended, in no small proportion, with the exciting ones of hope and love of life. Yet all these were resisted by activity of body and excitement of mind.

Many modern illustrations of this principle might be quoted, but our limits are restricted. The unheard-of privations, toils, and dangers survived by our own countrymen, the associates of Byron, through the wilds of South America, from Patagonia to Chili, offer a most splendid illustration of the principle in question. Xenophon's band marched through populous, though hostile countries, and it does not appear that thêy ever suffered much from hunger. Byron's small but determined crew were wasted to skeletons by want and cold—often compelled to eat grass and unwholesome berries, to allay the dreadful sensations of hunger.

> "In horrid climes, where Chiloe's tempests sweep
> The foaming surface of the tortur'd deep,
> 'Twas their's to mourn misfortune's rudest shock,
> Scourg'd by the winds, and cradled on the rock;—
> Yet, at thy call, the hardy tars pursued,
> (Pale but intrepid, sad but unsubdued)
> Pierced the deep woods, and hailing from afar
> The moon's pale planet and the northern star,
> Paus'd at each dreary cry, unheard before,
> Hyenas in the wild, and mermaids on the shore."

Through all this horrid series of dangers and woes, SICKNESS was absent, notwithstanding the operation of the most depressing passions co-operating with physical causes of disease. The ACTIVITY of the BODY seems here to have been the only principle of hygiene.

On a future occasion we shall enlarge upon this subject; in the mean time, the principle is applicable to private life as well as to national events —to individuals as well as to masses of men.

The interesting volume of Dr. Wilson, so replete with the most affecting incidents, will prove very useful, as well as amusing, to a large portion of English readers, because it embraces a description of the British settlements on the coasts of New Holland—more especially Raffles Bay, Melville Island, Swan River, King George's Sound, &c. It also contains important observations on transportation—the treatment of convicts during the voyage, and advice to persons intending to emigrate to the Australian colonies. To these may be added some highly amusing sketches of the manners and customs of the aboriginal tribes of the *New World*, to which Australia may lay claim, America being already antiquated.

